# Blood Pressure Management in Intracerebral Haemorrhage: when, how much, and for how long?

**DOI:** 10.1007/s11910-024-01341-2

**Published:** 2024-05-23

**Authors:** Chloe A. Mutimer, Nawaf Yassi, Teddy Y Wu

**Affiliations:** 1 Department of Medicine and Neurology, Melbourne Brain Centre at The Royal Melbourne Hospital, University of Melbourne, Parkville, 3050 Australia; 2 Population Health and Immunity Division, The Walter and Eliza Hall Institute of Medical Research, Parkville, 3052 Australia; 3 Department of Neurology, Christchurch Hospital, Christchurch, New Zealand; 4 Department of Medicine, University of Otago, Christchurch, New Zealand

**Keywords:** Intracerebral Haemorrhage, Hypertension, Antihypertensive Agents, Patient care Bundles

## Abstract

**Purpose of Review:**

When compared to ischaemic stroke, there have been limited advances in acute management of intracerebral haemorrhage. Blood pressure control in the acute period is an intervention commonly implemented and recommended in guidelines, as elevated systolic blood pressure is common and associated with haematoma expansion, poor functional outcomes, and mortality. This review addresses the uncertainty around the optimal blood pressure intervention, specifically timing and length of intervention, intensity of blood pressure reduction and agent used.

**Recent Findings:**

Recent pivotal trials have shown that acute blood pressure intervention, to a systolic target of 140mmHg, does appear to be beneficial in ICH, particularly when bundled with other therapies such as neurosurgery in selected cases, access to critical care units, blood glucose control, temperature management and reversal of coagulopathy.

**Summary:**

Systolic blood pressure should be lowered acutely in intracerebral haemorrhage to a target of approximately 140mmHg, and that this intervention is generally safe in the ICH population.

## Introduction

Intracerebral haemorrhage (ICH) accounts for 15–30% of stroke and has high 30-day mortality of approximately 40%, with only 12 to 39% of patients achieving independent functional recovery [1]. While there have been many recent advances in management of acute ischaemic stroke, there are currently very few treatments for ICH. Acute interventions in ICH have targeted haematoma expansion (HE), which occurs in up to one third of patients within the first 24 h and is strongly associated with neurological deterioration, poor functional outcome and mortality [1, 2]. Haemostatic therapy (recombinant factor VIIa [3, 4] or tranexamic acid [5, 6]) are not utilised in clinical practice with randomised trials showing variable effects on haematoma expansion, safety, functional outcome and mortality. The current mainstay of management in ICH remains supportive care in a stroke unit, reversal of anticoagulation if appropriate and acute blood pressure (BP) lowering. Surgical evacuation is typically reserved for selected patients with more severe ICH, particularly lobar, and can result in reduction of ICH volume and mass effect, and potential mitigation of secondary injury from iron toxicity and peri-haematomal oedema. The recently published Early Minimally Invasive Removal of Intracerebral Hemorrhage (ENRICH) trial is the only surgical evacuation trial to have shown benefit of haematoma evacuation on functional outcome (in the group with lobar haemorrhage) [7]. However, the optimal surgical technique is not known, with ongoing trials investigating minimally invasive techniques currently being undertaken. Other surgical procedures such as external ventricular drains and decompression, for significant hydrocephalus and posterior fossa bleeds, respectively, may be performed as life-saving procedures.

Hypertension on ICH presentation is common, and the main rationale for hypertensive therapy is to reduce haematoma expansion and therefore prevent further clinical deterioration and long-term poor functional outcomes [8••]. However, inconsistent results amongst clinical trials [9••, 10••, 11] have led to weak recommendations in guidelines regarding acute blood pressure therapy [1, 12–14]. Controversies persist over the target, timing, intensity and agents used. This review aims to summarise the recent literature and current guidelines regarding acute blood pressure lowering in ICH.

## When?

The principle of “time is brain” from ischaemic stroke has driven interest in early blood pressure lowering in ICH, particularly given that the rate of haematoma expansion is highest in the first 3 h and the amount of haematoma expansion increases the odds of dependence or death [8••]. In the last decade, there have been two randomised clinical trials and one other international cluster-randomised trial that have investigated early, intensive blood pressure control on long-term clinical outcomes in ICH (Table 1). Both the INTERACT2 (Intensive blood pressure Reduction in Acute Cerebral Haemorrhage Trial) and ATACH-2 (Antihypertensive Treatment of Acute Cerebral Haemorrhage) trials aimed for a reduction in systolic blood pressure (SBP) for patients presenting within 6 h and 4.5 h of symptom onset respectively, but included on average patients with mild-moderate severity ICH with relatively small baseline haematoma volume [9••]. INTERACT2 aimed for blood pressure to be at target after 1 h. Both trials did not meet their primary outcome of improving functional outcome at 90-days, although INTERACT2 showed a positive effect on ordinal mRS analysis (pooled OR = 0.87, 95% CI 0.77-1.00, *p* = 0.04). There was no clear benefit found for those randomised in the early time window (< 4 h) compared to a late time window (≥ 4 h). Additionally, neither trial showed a statistically significant effect on reducing haematoma expansion. A post-hoc analysis of ATACH-2 did show benefit in achieving functional independence (modified Rankin Scale [mRS] 0–2) and reducing haematoma expansion in the ultra-early (2 h) and “fast-bleeding” (haematoma growth > 5 ml/hr) treatment groups [15, 16]. Current best practice guideline recommendations are based on these trials, with American Heart Association/American Stroke Association and European Stroke Organisation guidelines providing weak recommendations with the aim to institute blood pressure lowering within 2 h of symptom onset (Table 2). There are, however, no time specific quality metrics, similar to door-to-needle time in acute ischaemic stroke, that are specified for care of ICH patients.

**Table 1:** summary of large multicentre trials relating to blood pressure control in ICH

	INTERACT2 [9••]	ATACH-2 [10••]	INTERACT3 [11•]
Trial type	International, multicentre, prospective, randomised, open-treatment, blinded end-point trial	International, multicentre, prospective, randomised, two-group, open-label trial	International, multicentre, prospective, stepped wedge, cluster randomised, blinded, outcome assessed, controlled trial
Year published	2013	2016	2023
Intervention	Target SBP ≤ 140mmHg within 1 h	Target SBP 110-139mmHg	Bundle of care: target SBP ≤ 140mmHg within 1 h, BSL target (non-diabetic patients 6.1-7.8mmol/L, diabetic patients 7.8-10.0mmol/L), temperature ≤ 37.5 degrees within 1 h, reversal of abnormal coagulation within 1 h
Length of intervention	7 days	24 h	7 days
Patients in intervention arm	1399	500	3221
Inclusion criteria	Age ≥ 18 years Primary ICH SBP 150-220mmHg 6 h of symptom onset GCS ≥ 6 Massive haematoma with poor prognosis Surgical management planned	Age ≥ 18 years Primary ICH SBP ≥ 180mmHg 4.5 h of symptom onset GCS ≥ 5 Intraparenchymal haematoma volume ≤ 60 ml	Age ≥ 18 years Primary ICH SBP 150-220mmHg 6 h of symptom onset
Primary outcome	mRS 3–6 at 90 days	mRS 4–6 at 90 days	Ordinal mRS at 6 months
Baseline characteristics in intervention groups			
Median age (years)	63.0 ± 13.1	62.0 ± 13.1	61.8 ± 12.6
Male sex (%)	64.2	60.8	64.4
Mean systolic blood pressure (mmHg)	179.0 ± 17.0	200.0 ± 27.1	174.9 ± 28.2
Mean diastolic blood pressure (mmHg)	101.0 ± 15.0	Not reported	99.7 ± 17.7
Median NIHSS (IQR)	14 (12–15)	11 (0–40)	13 (7.23)
Median GCS score (IQR)	14 (12–15)	Not reported	12 (9–14)
History of hypertension (%)	72.4	82.2	68.5
Use of anticoagulation (%)	3.6	Not reported	0.9
Use of antiplatelets (%)	8.8	Not reported	4.7
Median ICH volume, mL	11.0 (IQR 6–19)	10.3 (range 2.3–85.2)	15.0 (IQR 7–30)
Deep location of haematoma (%)	83.8	90.3	83.9
Intraventricular extension (%)	28.7	24.6	26.8
Treatment characteristics in intervention groups			
Time from symptom to start of treatment, hours	Median 4.0 (IQR 2.9–5.1)	Mean 2.5 ± 1.1	Not reported
Use of intravenous agent (%)	90.1	100.0	78.9
Clinical and safety outcomes in intervention groups			
Primary outcome	mRS 3–6 at 90 days = 52.0% (*p* = 0.06 compared to control group)	mRS 4–6 at 90 days = 38.7% (*p* = 0.84 compared to control group)	Ordinal mRS: OR 0.86 (95% CI 0·76–0·97; *p* = 0·015)
Mean systolic blood pressure post-randomisation (mmHg)	150 at 1 h	128.9 ± 16 at 2 h	148.4 ± 21.5 at 1 h
Mortality (%)	11.9 (*p* = 0.96 compared to control group)*	6.6 (*p* = 0.90 compared to control group)^†^	14.1^‡^
Neurological deterioration at 24 h	14.5% (*p* = 0.62 compared to control group)^§^	11.0% (*p* = 0.13 compared to control group)^¶^	Not reported
Hypotension ^#^	0.5% (*p* = 0.49 compared to control group)^#^	1.2% (0.33 compared to control group)^†^	Not reported
Haematoma expansion	35.1% (*p* = 0.27 compared to control group)^**^	18.9% (unadjusted *p* = 0.09 compared to control group)^††^	Not reported
Comments			
Strengths	- Multi-centre trial - Pertinent safety outcomes reported	- Multi-centre trial - Standardised use of antihypertensives - Pertinent safety outcomes reported	- Multi-centre trial - Large sample size - More severe ICH included compared to INTERACT2 and ATACH-2
Limitations	- Mild-moderate ICH severity only included - No standardised agent used - Unblinded to intervention	- Mild-moderate ICH severity only included - Underpowered for primary outcome - Inclusion criteria changed mid-trial - BP control could be instituted pre-randomisation	- Not an RCT (“gold standard”) - Generalisability to high income countries unclear - No standardised agent used

^*^At 90 days

^†^At 6 months

^‡^Within 72 h

^§^As defined by any clinician-reported neurological deterioration of NIHSS ≥ 4 points from baseline, cerebral mass effect or haematoma extension

^¶^As defined by a Glasgow Coma Scale decrease of ≥ 2 points from baseline, or an increase NIHSS ≥ 4 points from baseline

^#^Hypotension with clinical consequence requiring corrective therapy with intravenous fluids, vasopressors or haemodialysis

^**^Any haematoma growth from baseline

^††^An increase of 33% or more in the haematoma volume from baseline to 24 h

**Table 2:** current stroke guideline recommendations regarding acute blood pressure management in ICH

	When?	How much?	How long?
American Heart Association/American Stroke Association [1]	Within 2 h of onset, ideally at target within 1 h *Class 2a*	SBP 140mmHg (range 130-150mmHg) in patients with mild-moderate ICH presenting with SBP 150-220mmHg *Class 2b*	No recommendation
European Stroke Organisation [12]	As early as possible, ideally within 2 h *Expert consensus*	SBP 140mmHg (and above 110mmHg) if presenting within 6 h *Weak recommendation*	24–72 h *Expert consensus*
Chinese Stroke Association [13]	No recommendation	SBP 140mmHg in patients with SBP > 150mmHg *Class 2b*	No recommendation
Australian Stroke Foundation [14]	No recommendation	Less than 140mmHg, but not substantially lower *Weak recommendation*	No recommendation

More recently, bundled care for ICH, incorporating control of BP, blood glucose, temperature and correction of coagulopathy has been supported by the INTERACT3 trial [11•], with the primary outcome of ordinal mRS at 6 months improved in the intervention arm (OR = 0.86, 95% CI 0.76–0.97; *p* = 0.015). Patients were eligible if randomised within 6 h of symptom onset and the target systolic blood pressure was less than 140mmHg within 1 h of treatment commencement, although the actual median time to reach target was 2.3 h (IQR 0.8–8.0 h). In contrast to INTERACT2 and ATACH-2, severe ICH patients were included. Given the trial design, it is difficult to know how much benefit stemmed from blood pressure control alone. The implementation of bundled care and quality metrics, has been further supported by an expert consensus statement in the *European Stroke Journal* [17] and a “Code ICH” article in *Stroke* [18] with a proposed door-to-first antihypertensive time of ≤ 30 min in the former, and a door-to-target time of ≤ 60 min in both articles.

The role of pre-hospital blood pressure management has also been explored. RIGHT-2 (Prehospital transdermal glyceryl trinitrate in patients with ultra-acute presumed stroke) and MR ASAP (Prehospital transdermal glyceryl trinitrate in patients with presumed acute stroke) were both ambulance-based trials of topical nitrates for presumed acute stroke (ischaemic and haemorrhagic) with neutral overall outcomes (ordinal mRS at 90 days). Additionally, there was suggestion of harm in ICH in both trials, which lead to early termination of MR ASAP [19, 20]. Given these findings, management of hypertension in suspected stroke in the pre-hospital setting is not recommended.

The advent of mobile stroke units allows for ultra-early diagnosis and intervention for all stroke types, including blood pressure control in ICH [21]. This has been formally investigated in a sub-study of the B_PROUD (Berlin_Prehospital Or Usual Care Delivery in acute Stroke) study [22], where ICH patients were prospectively evaluated and compared to patients seen by conventional ambulance. This study had small patient numbers and was a neutral study, although the primary endpoint was mortality at 7 days (aOR = 1.43, 95% CI 0.68–3.31), rather than functional outcome. It was shown that systolic blood pressure was lower for the MSU cohort on hospital arrival (161mmHg vs. 177mmHg), but there was no improvement in the secondary endpoint of mRS ≥ 3 outcome (aOR = 1.21, 95% CI 0.56–2.61). Further data is needed to determine the efficacy of ultra-early blood pressure management for ICH in the mobile stroke unit setting.

## How much?

All three large clinical trials had very similar systolic blood pressure targets, aiming less than 140mmHg, with a lower limit of systolic blood pressure 110mmHg specified in ATACH-2 [9••, 10••, 11]. The mean systolic blood pressure at 2 h in ATACH-2 was 128.9mmHg (compared to 141.1mmHg in the control group), with 12.2% not meeting target at 24 h. Systolic blood pressure at 1 h in the intervention group was similar in both the INTERACT2 (150mmHg vs. 164mmHg) and INTERACT3 trials (148.4mmHg vs. 154.7mmHg). While only INTERACT3 was a positive study, acute blood pressure lowering to this level was found to be generally safe in all 3 trials, without a significant increase in safety outcomes including mortality or pertinent other adverse outcomes, such as severe hypotension, recurrent ischaemic stroke and serious renal impairment. There were increased rates of any renal impairment within 7 days seen in intervention arm of ATACH-2 (aOR 2.32, 95% CI 1.37–3.94, *p* = 0.0018). These systolic blood pressure targets have additionally been shown to be safe in patients undergoing surgical haematoma evacuation, a more severe subgroup of patients [23].

Large systolic blood pressure drops should be avoided, as shown in a pooled analysis of patients from the INTERACT2 and ATACH-2 trials (predominantly mild-to-moderate severity I, where systolic blood pressure drops of > 60mmHg within the first hour were found to be harmful [24]. Hypotension (i.e. a systolic blood pressure of less than 100mmHg) is avoided in clinical practice, and on presentation with ICH has been shown to be associated with poor outcome [25]. Given this evidence, there is consensus amongst guidelines to target a systolic blood pressure of 140mmHg, rather than less than 140mmHg, accepting that this may be practically challenging. One concern of aggressive blood pressure lowering is secondary ischaemic injury, which can manifest as remote ischaemic lesions in acute ICH [26]. However, the post hoc analysis of ATACH-2 and a large observational study (Ethnic/Racial Variations of Intracerebral Hemorrhage, ERICH) did not demonstrate an association with intensive blood pressure lowering and risk of new ischaemic injury [27, 28].

All trials have focused on systolic blood pressure as the primary metric. Other blood pressure metrics, primarily systolic blood pressure variability, have been explored. Systolic blood pressure variability has been shown to be associated with poor functional outcomes in ICH, and it has been suggested that sustained control, with avoidance of peaks, may enhance the benefits of blood pressure reduction [29, 30]. The effect on haematoma expansion and functional outcomes in ICH when targeting diastolic blood pressure and mean arterial blood pressure are not well explored in the literature, with one small randomised study that targeted a mean arterial pressure less than 115mmHg, showing no improvement in functional outcomes [31]. Given this, there are no recommendations relating to diastolic or mean arterial pressure targets in the guidelines.

## How long?

The optimal length of acute blood pressure intervention in ICH is not known, relating to the paucity of high-quality data. The length of intervention in INTERACT2 and INTERACT3 was 7 days, and 24 h in ATACH-2 [9••, 10••, 11]. Given this, only the European Stroke Organisation guidelines comment on length of acute blood pressure management (24–72 h) which was an expert consensus decision. Given that most haematoma expansion occurs within the first few hours after ICH, it may be practical to aim for the same systolic blood pressure target (less than 140mmHg), however with less aggressive interventions (i.e. oral rather than intravenous medications) after the first 24 h.

After the acute period, long-term blood pressure management should be instituted to prevent recurrent ischaemic and haemorrhagic stroke. Several randomised controlled trials have investigated the optimal long-term target and agent of choice in the prevention of recurrent stroke [32–34], with varying results, however all supporting blood pressure management in preventing recurrent stroke. International Society of Hypertension/American Heart Association guidelines recommend targeting a systolic blood pressure of less than 130-140mmHg depending on patient age, with a target of less than 130/80mmHg recommended in the European guidelines, with low-dose combination therapy initially [35, 36]. We suggest leniency in terms of targets after the 24-hour time period, actively intervening on a systolic blood pressure above 180mmHg, with gradual up-titration of oral agents as a first step, depending on blood pressure trend.

All large trials recruited patients within 4.5–6 h from their symptom onset, and therefore aggressive blood pressure lowering with intravenous agents in patients presenting after 24 h from symptom onset is likely of limited benefit. We suggest that patients with late presentations be managed with oral medication with gradual blood pressure reduction to long-term targets.

## Which agent?

Intravenous agents are typically preferred given conscious state and dysphagia concerns in many ICH patients acutely. However, the optimal agent or class of drugs to control blood pressure is not known and has not been well explored in trials. INTERACT2 and INTERACT3 allowed sites to choose their preferred agent [9••, 11]. Given this, local availability and practices affected medication choice, and alpha-adrenergic antagonists, such as uradipil (> 60% in INTERACT3), were the most common agents used, a medication rarely used in other settings. Intravenous nicardipine was the first-line agent in the ATACH-2 trial (with intravenous labetalol second-line) and in an individual participant data systemic review of this trial and 2 additional trials, 24 h of nicardipine use to control blood pressure in the hyperacute period was shown to result in rapid lowering of systolic blood pressure with associations with reduced haematoma expansion and better functional outcomes [37]. From a practical perspective, nicardipine is a dihydropyridine-derived calcium channel blocker which is rapidly titratable, with limited adverse effects [38]. Other commonly used agents include, hydralazine (a hydrazine derivative vasodilator), which can be associated with reflex tachycardia and tachyphylaxis [39], and labetalol (a beta-blocker) with its use limited by bradyarrhythmias and bronchospasm [38]. A cohort study investigated the use of intravenous hydralazine, labetalol and nicardipine, and found agent used was associated with only initial reduction in diastolic but not systolic BP, and there were no differences in subsequent clinical outcomes [40].

In terms of non-intravenous options, transdermal glyceryl trinitrate has typically been avoided due to the outcomes of the previously discussed RIGHT-2 (trend toward harm in ICH) and MR ASAP trials [19, 20]. In MR ASAP, of patients with ICH, mortality at 7 days was 34% in the intervention arm compared to 10% in the control arm (aOR = 5.91, 95% CI 0.78–44.81), with similar findings at 90 days. It has been postulated that glyceryl trinitrate causes harm in ICH due amelioration of protective vasoconstriction to prevent haematoma expansion, and thus other administration options of glyceryl trinitrate, including intravenous, have been avoided [41].

Practically, once patients are safe to swallow, oral medication should be instituted. A return to an intravenous infusion could be considered in patients with refractory hypertension after the first 24 h (as an example, a sustained systolic blood pressure above 180mmHg) despite combination oral therapy. No guidelines make specific recommendations in relation to agent of choice, but ultimately local availability, practicality, pharmacological profile, patient parameters (such as renal function and heart rate), potential adverse effects and cost should be considered.

## Recommendations

In all patients presenting with acute ICH (regardless of severity of stroke syndrome), we recommend that institution of blood pressure management should be as early as possible, aiming for a target systolic blood pressure of approximately 140mmHg, but not substantially below, with a target range of 120-140mmHg. The intensity to which the blood pressure is lowered to this target depends on several factors such as initial systolic blood pressure, frailty and renal function. Given its efficacy, tolerability, and practicality, intravenous nicardipine should be considered the first line agent in the acute period where available. Acute blood pressure management should be provided in a bundle, incorporating anticoagulation reversal and correction of hyperglycaemia and pyrexia. Our practice is to avoid admission to the intensive care unit for the sole purpose of blood pressure management, but we acknowledge there is variability in monitoring and intravenous drug options available on stroke units. After the first 24 h, it is practical to aim for the same systolic blood pressure target (≤ 140mmHg), however with switch to enteral combination therapy when safe. In order to improve overall care for ICH patients in the acute period, quality time metrics could be established and monitored in a similar paradigm to ischaemic stroke.

## Conclusions

While there is consensus amongst major stroke organisation guidelines relating to the numeric target for blood pressure control, these recommendations are weak, and there remains discrepancies and controversies in the literature relating to the target, timing, speed, and agents use. This is primarily underpinned by conflicting data and the lack of recent randomised controlled trials in this field. Importantly, there is a large body of evidence supporting the safety of blood pressure control in ICH. Bundled care, including blood pressure control, has been shown to be efficacious in a broad and real-world patient population and should be instituted in all facilities caring for patients with ICH. Further evidence is needed to support current blood pressure targets in acute ICH, with future directions including investigation of optimal agent, and impact of other blood pressure parameters (such as variability or mean arterial pressure).

## Data Availability

No datasets were generated or analysed during the current study.

## Declarations

### Statements and Declarations

CM is supported by an Australian Government Research Training Program Scholarship and The University of Melbourne Rowden White Scholarship.
